# Supplementary material for: A Likelihood Approach for Real-Time Calibration of Stochastic Compartmental Epidemic Models
Source: PLoS Comput Biol. 2017 Jan 17;13(1):e1005257. doi: 10.1371/journal.pcbi.1005257 (PMC5240920; doi:10.1371/journal.pcbi.1005257)
Supplement: S1 File — (TAR.GZ) [file pcbi.1005257.s014.tar.gz › HSPH_Online-SI-Revision/PF08/n10-extreme/n10-extreme_PF2_table-4.pdf]

1	newly 3 weeks spec	1350.	386.46	{197., 643.}	71.3733	0.58501	newly 3 weeks cumul	2770.	959.88	{502., 1496.}	65.3473	0.497051	newly 5 weeks spec	1264.	477.03	{243., 818.}	62.2603	0.462158	newly 5 weeks cumul	5455.	1880.39	{970., 3013.}	65.5291	0.499925	newly 62 weeks cumul	8628.	6527.42	{4582., 8281.}	24.3611	0.131455
2	newly 3 weeks spec	604.	363.35	{238., 477.}	39.8427	0.235487	newly 3 weeks cumul	1548.	977.25	{635., 1284.}	36.8702	0.212931	newly 5 weeks spec	678.	400.54	{268., 519.}	40.9233	0.242372	newly 5 weeks cumul	2970.	1761.15	{1164., 2318.}	40.702	0.239878	newly 58 weeks cumul	6502.	5833.64	{4660., 6633.}	11.4688	0.056636
3	newly 3 weeks spec	1586.	410.94	{121., 706.}	74.0895	0.667408	newly 3 weeks cumul	3187.	950.26	{298., 1590.}	70.1832	0.594547	newly 5 weeks spec	1325.	553.89	{156., 918.}	58.197	0.460173	newly 5 weeks cumul	6258.	1999.02	{583., 3382.}	68.0566	0.570063	newly 64 weeks cumul	8818.	7168.65	{5269., 8770.}	19.1591	0.100806
4	newly 3 weeks spec	888.	487.5	{373., 623.}	45.1014	0.269032	newly 3 weeks cumul	2367.	1346.14	{1068., 1718.}	43.1289	0.252922	newly 5 weeks spec	862.	492.33	{367., 609.}	42.8852	0.250794	newly 5 weeks cumul	4124.	2333.59	{1811., 2937.}	43.4144	0.254631	newly 58 weeks cumul	7204.	5911.22	{4769., 6777.}	17.9453	0.0905356
5	newly 3 weeks spec	1149.	708.77	{467., 935.}	38.3142	0.224298	newly 3 weeks cumul	2806.	1837.81	{1199., 2456.}	34.5043	0.198316	newly 5 weeks spec	1043.	725.23	{482., 903.}	30.4669	0.168994	newly 5 weeks cumul	5000.	3307.66	{2222., 4266.}	33.8468	0.192112	newly 61 weeks cumul	8544.	7328.8	{5602., 8426.}	14.2282	0.0716321
6	newly 3 weeks spec	1137.	480.05	{203., 743.}	57.7792	0.424789	newly 3 weeks cumul	2504.	1183.3	{513., 1826.}	52.7436	0.370673	newly 5 weeks spec	1175.	578.62	{257., 900.}	50.7557	0.352411	newly 5 weeks cumul	4971.	2308.3	{1058., 3528.}	53.5647	0.377818	newly 62 weeks cumul	8740.	6965.11	{4651., 8556.}	20.3255	0.107461
7	newly 3 weeks spec	962.	442.61	{206., 739.}	54.1154	0.385549	newly 3 weeks cumul	2111.	1072.94	{521., 1743.}	49.7016	0.340304	newly 5 weeks spec	1027.	550.62	{249., 863.}	46.4362	0.313039	newly 5 weeks cumul	4213.	2134.24	{995., 3426.}	49.5201	0.338977	newly 63 weeks cumul	7880.	6947.17	{4864., 8284.}	14.3773	0.0726296
8	newly 3 weeks spec	963.	416.66	{280., 574.}	56.7331	0.383056	newly 3 weeks cumul	2334.	1083.51	{699., 1452.}	53.5771	0.351205	newly 5 weeks spec	978.	483.35	{320., 638.}	50.5777	0.321269	newly 5 weeks cumul	4380.	2022.72	{1343., 2695.}	53.8192	0.352069	newly 60 weeks cumul	8557.	6492.12	{4985., 7421.}	24.1309	0.124463
9	newly 3 weeks spec	987.	486.72	{305., 643.}	50.6869	0.327346	newly 3 weeks cumul	2373.	1287.57	{818., 1795.}	45.7408	0.285669	newly 5 weeks spec	932.	522.63	{321., 689.}	43.9238	0.267691	newly 5 weeks cumul	4333.	2325.93	{1440., 3212.}	46.3206	0.288601	newly 60 weeks cumul	8041.	6420.42	{5112., 7546.}	20.154	0.102805
10	newly 3 weeks spec	784.	398.29	{255., 545.}	49.1977	0.310374	newly 3 weeks cumul	1916.	1044.65	{711., 1411.}	45.4776	0.278968	newly 5 weeks spec	979.	455.94	{310., 590.}	53.428	0.34511	newly 5 weeks cumul	3879.	1933.16	{1345., 2559.}	50.1634	0.316771	newly 60 weeks cumul	8180.	6315.41	{5254., 7150.}	22.7945	0.115784
11	newly 3 weeks spec	908.	319.38	{265., 379.}	64.826	0.458255	newly 3 weeks cumul	2169.	901.94	{759., 1052.}	58.4168	0.384704	newly 5 weeks spec	841.	330.68	{270., 402.}	60.6801	0.409974	newly 5 weeks cumul	3950.	1562.08	{1321., 1847.}	60.4537	0.406477	newly 55 weeks cumul	7949.	5256.42	{4827., 5764.}	33.8732	0.180663
12	newly 3 weeks spec	811.	315.7	{183., 439.}	61.0727	0.430627	newly 3 weeks cumul	1803.	813.33	{492., 1102.}	54.8902	0.363319	newly 5 weeks spec	1021.	382.71	{213., 539.}	62.5162	0.448053	newly 5 weeks cumul	3891.	1548.	{914., 2136.}	60.2159	0.419359	newly 60 weeks cumul	7914.	6205.25	{5022., 7221.}	21.5915	0.110004
13	newly 3 weeks spec	841.	440.6	{323., 563.}	47.61	0.289462	newly 3 weeks cumul	2317.	1251.59	{908., 1603.}	45.9823	0.275841	newly 5 weeks spec	848.	437.53	{330., 522.}	48.4045	0.293491	newly 5 weeks cumul	4080.	2136.	{1568., 2668.}	47.6471	0.288356	newly 56 weeks cumul	7098.	5643.23	{4738., 6185.}	20.4955	0.10186
14	newly 3 weeks spec	971.	677.21	{446., 897.}	30.7446	0.175133	newly 3 weeks cumul	2497.	1766.78	{1206., 2266.}	29.6452	0.167216	newly 5 weeks spec	946.	701.24	{499., 878.}	26.167	0.142921	newly 5 weeks cumul	4507.	3179.95	{2186., 4151.}	29.5511	0.165394	newly 61 weeks cumul	7771.	7194.6	{5942., 8217.}	10.7402	0.0511169
15	newly 3 weeks spec	832.	444.36	{329., 528.}	46.5913	0.280268	newly 3 weeks cumul	2173.	1214.21	{877., 1415.}	44.1229	0.260191	newly 5 weeks spec	838.	467.47	{338., 549.}	44.216	0.260595	newly 5 weeks cumul	3908.	2142.42	{1560., 2494.}	45.1786	0.267898	newly 58 weeks cumul	6416.	5897.09	{4786., 6616.}	9.80969	0.0474956
16	newly 3 weeks spec	1062.	356.87	{193., 593.}	66.3964	0.507532	newly 3 weeks cumul	2353.	910.06	{495., 1485.}	61.3234	0.443631	newly 5 weeks spec	1082.	433.78	{234., 695.}	59.9094	0.427423	newly 5 weeks cumul	4527.	1745.58	{952., 2864.}	61.4407	0.444496	newly 61 weeks cumul	8363.	6533.32	{5088., 7701.}	21.9089	0.11202
17	newly 3 weeks spec	1033.	522.34	{206., 829.}	49.6341	0.356873	newly 3 weeks cumul	2323.	1254.27	{531., 1955.}	46.6513	0.323203	newly 5 weeks spec	1116.	630.57	{261., 948.}	43.4973	0.297939	newly 5 weeks cumul	4627.	2478.24	{984., 3858.}	46.5675	0.323656	newly 63 weeks cumul	8302.	7195.73	{4953., 8711.}	15.5051	0.08011
18	newly 3 weeks spec	1151.	534.41	{318., 765.}	53.5699	0.358102	newly 3 weeks cumul	2756.	1364.99	{874., 1966.}	50.4721	0.327796	newly 5 weeks spec	1119.	595.4	{366., 841.}	46.7918	0.294641	newly 5 weeks cumul	5058.	2539.76	{1582., 3569.}	49.7873	0.320739	newly 61 weeks cumul	8663.	6934.65	{5371., 8130.}	19.9509	0.102191
19	newly 3 weeks spec	1098.	314.11	{123., 596.}	71.3925	0.610598	newly 3 weeks cumul	2182.	749.24	{321., 1300.}	65.6627	0.521585	newly 5 weeks spec	1350.	425.73	{152., 846.}	68.4644	0.571246	newly 5 weeks cumul	4959.	1554.3	{613., 2955.}	68.657	0.56728	newly 63 weeks cumul	8373.	6637.33	{4729., 8627.}	21.9753	0.115723
20	newly 3 weeks spec	902.	396.39	{242., 553.}	56.0543	0.378943	newly 3 weeks cumul	2235.	1043.42	{660., 1403.}	53.3145	0.350408	newly 5 weeks spec	986.	454.89	{294., 614.}	53.8651	0.353386	newly 5 weeks cumul	4273.	1927.71	{1201., 2622.}	54.8863	0.363731	newly 60 weeks cumul	8476.	6238.15	{4762., 7407.}	26.4022	0.138964
21	newly 3 weeks spec	966.	400.24	{280., 528.}	58.5673	0.395172	newly 3 weeks cumul	2273.	1115.73	{816., 1478.}	50.9138	0.321274	newly 5 weeks spec	836.	421.7	{312., 542.}	49.5574	0.306875	newly 5 weeks cumul	4121.	1954.85	{1444., 2523.}	52.5637	0.335	newly 57 weeks cumul	7668.	5890.78	{4943., 6530.}	23.1771	0.117065
22	newly 3 weeks spec	1428.	287.32	{103., 512.}	79.8796	0.781772	newly 3 weeks cumul	2568.	658.41	{248., 1213.}	74.361	0.66275	newly 5 weeks spec	1622.	426.58	{128., 825.}	73.7004	0.673611	newly 5 weeks cumul	6049.	1448.79	{517., 2581.}	76.0491	0.701651	newly 64 weeks cumul	9138.	6966.43	{4992., 8870.}	23.8491	0.128645
23	newly 3 weeks spec	671.	451.67	{318., 603.}	33.0507	0.187547	newly 3 weeks cumul	1760.	1213.89	{826., 1581.}	32.179	0.180262	newly 5 weeks spec	667.	485.33	{334., 653.}	28.4333	0.156354	newly 5 weeks cumul	3172.	2171.54	{1555., 2787.}	32.1513	0.180348	newly 59 weeks cumul	5972.	6181.07	{4950., 7051.}	12.5052	0.0544598
24	newly 3 weeks spec	922.	512.67	{393., 607.}	44.3959	0.259701	newly 3 weeks cumul	2515.	1460.9	{1173., 1778.}	41.9125	0.240743	newly 5 weeks spec	778.	489.9	{408., 563.}	37.0308	0.204556	newly 5 weeks cumul	4256.	2459.	{1969., 2998.}	42.2227	0.242353	newly 57 weeks cumul	7579.	5792.79	{4762., 6381.}	23.5679	0.119363
25	newly 3 weeks spec	1376.	278.16	{163., 388.}	79.7849	0.718358	newly 3 weeks cumul	2783.	714.8	{443., 991.}	74.3155	0.610745	newly 5 weeks spec	1327.	340.2	{193., 481.}	74.3632	0.616251	newly 5 weeks cumul	5644.	1368.09	{821., 1851.}	75.7603	0.63722	newly 60 weeks cumul	8660.	6290.02	{5371., 7241.}	27.367	0.141883
26	newly 3 weeks spec	1193.	511.97	{241., 769.}	57.0855	0.414077	newly 3 weeks cumul	2728.	1224.29	{566., 1866.}	55.1213	0.38938	newly 5 weeks spec	913.	637.12	{263., 940.}	32.506	0.209185	newly 5 weeks cumul	4845.	2458.45	{1154., 3638.}	49.258	0.336578	newly 63 weeks cumul	6836.	7259.69	{5286., 8688.}	15.6965	0.0668939
27	newly 3 weeks spec	1034.	356.23	{214., 556.}	65.5484	0.493796	newly 3 weeks cumul	2307.	901.9	{542., 1382.}	60.9059	0.436284	newly 5 weeks spec	1086.	432.14	{266., 676.}	60.2081	0.42758	newly 5 weeks cumul	4510.	1735.87	{1085., 2615.}	61.5106	0.442019	newly 61 weeks cumul	8570.	6464.49	{5126., 7568.}	24.5684	0.127189
28	newly 3 weeks spec	1424.	463.04	{126., 857.}	67.4831	0.581438	newly 3 weeks cumul	2886.	1076.99	{345., 1942.}	62.6823	0.510272	newly 5 weeks spec	1362.	599.76	{189., 1034.}	55.9648	0.439951	newly 5 weeks cumul	5761.	2220.64	{671., 3934.}	61.4539	0.496222	newly 64 weeks cumul	8981.	7220.08	{5075., 8950.}	19.8152	0.105707
29	newly 3 weeks spec	1269.	340.69	{221., 452.}	73.1529	0.589424	newly 3 weeks cumul	2814.	891.92	{579., 1160.}	68.3042	0.514927	newly 5 weeks spec	1154.	389.52	{253., 514.}	66.2461	0.488023	newly 5 weeks cumul	5251.	1650.42	{1084., 2128.}	68.5694	0.518377	newly 59 weeks cumul	8475.	6049.03	{4971., 6964.}	28.625	0.151238
30	newly 3 weeks spec	975.	400.49	{257., 523.}	58.9241	0.402741	newly 3 weeks cumul	2275.	1059.56	{675., 1359.}	53.4259	0.348415	newly 5 weeks spec	882.	434.99	{280., 568.}	50.6814	0.321836	newly 5 weeks cumul	4197.	1921.19	{1211., 2472.}	54.2247	0.354918	newly 59 weeks cumul	7393.	6161.3	{4928., 7020.}	16.6604	0.0829396
31	newly 3 weeks spec	1130.	592.82	{282., 845.}	47.5646	0.30936	newly 3 weeks cumul	2628.	1486.28	{736., 2073.}	43.7154	0.276081	newly 5 weeks spec	1066.	663.31	{370., 885.}	37.7758	0.226601	newly 5 weeks cumul	4862.	2795.23	{1390., 3837.}	42.5202	0.264934	newly 62 weeks cumul	8496.	7113.81	{5483., 8370.}	16.4528	0.0836497
32	newly 3 weeks spec	826.	343.11	{272., 409.}	58.4613	0.386694	newly 3 weeks cumul	2183.	990.14	{793., 1165.}	54.6432	0.347929	newly 5 weeks spec	784.	340.89	{272., 404.}	56.5191	0.365864	newly 5 weeks cumul	3849.	1677.43	{1367., 1936.}	56.4191	0.36484	newly 54 weeks cumul	7242.	5084.83	{4647., 5497.}	29.7869	0.154734
33	newly 3 weeks spec	1646.	407.27	{185., 679.}	75.257	0.659865	newly 3 weeks cumul	3764.	1012.41	{477., 1667.}	73.1028	0.617528	newly 5 weeks spec	1179.	492.26	{199., 798.}	58.2477	0.428884	newly 5 weeks cumul	6592.	1966.18	{859., 3166.}	70.1732	0.574151	newly 62 weeks cumul	8743.	6437.97	{4623., 8172.}	26.3643	0.143317
34	newly 3 weeks spec	1346.	653.32	{302., 919.}	51.4621	0.350601	newly 3 weeks cumul	2950.	1528.73	{810., 2171.}	48.1786	0.318197	newly 5 weeks spec	1080.	768.25	{388., 1031.}	29.1194	0.179767	newly 5 weeks cumul	5397.	3038.6	{1540., 4243.}	43.6984	0.281459	newly 64 weeks cumul	7875.	7622.46	{5488., 8913.}	12.5531	0.0590622
35	newly 3 weeks spec	787.	477.33	{322., 611.}	39.3482	0.228014	newly 3 weeks cumul	2046.	1289.59	{930., 1652.}	36.979	0.211466	newly 5 weeks spec	782.	508.31	{358., 619.}	34.9987	0.196464	newly 5 weeks cumul	3686.	2302.26	{1645., 2909.}	37.5404	0.214554	newly 59 weeks cumul	7161.	6330.38	{5128., 7037.}	11.9092	0.0585126
36	newly 3 weeks spec	900.	381.	{258., 513.}	57.6667	0.386945	newly 3 weeks cumul	2289.	1050.88	{748., 1435.}	54.09	0.351491	newly 5 weeks spec	946.	398.81	{293., 509.}	57.8425	0.384884	newly 5 weeks cumul	4201.	1844.91	{1335., 2448.}	56.084	0.369267	newly 57 weeks cumul	8382.	5840.06	{4919., 6571.}	30.3262	0.159462
37	newly 3 weeks spec	1140.	447.25	{247., 674.}	60.7675	0.430129	newly 3 weeks cumul	2692.	1160.18	{658., 1740.}	56.9027	0.388221	newly 5 weeks spec	1097.	506.45	{310., 710.}	53.8332	0.354265	newly 5 weeks cumul	4963.	2153.75	{1289., 3097.}	56.6039	0.38312	newly 60 weeks cumul	8552.	6549.19	{5411., 7370.}	23.4192	0.119563
38	newly 3 weeks spec	1819.	293.42	{121., 572.}	83.8692	0.856498	newly 3 weeks cumul	3439.	711.15	{328., 1264.}	79.321	0.737747	newly 5 weeks spec	1286.	399.99	{163., 787.}	68.8966	0.576475	newly 5 weeks cumul	6558.	1465.55	{616., 2810.}	77.6525	0.711201	newly 63 weeks cumul	8641.	6557.86	{4830., 8493.}	24.4203	0.129741
39	newly 3 weeks spec	1549.	456.86	{198., 759.}	70.5061	0.582225	newly 3 weeks cumul	3391.	1082.45	{505., 1874.}	68.0787	0.545178	newly 5 weeks spec	1272.	581.84	{244., 904.}	54.2579	0.385372	newly 5 weeks cumul	6224.	2198.07	{939., 3628.}	64.684	0.499862	newly 63 weeks cumul	8953.	7266.4	{5669., 8560.}	18.844	0.0961351
40	newly 3 weeks spec	954.	555.56	{342., 766.}	41.7652	0.254077	newly 3 weeks cumul	2231.	1483.97	{933., 2038.}	33.511	0.195271	newly 5 weeks spec	887.	586.51	{380., 780.}	33.8771	0.19429	newly 5 weeks cumul	4133.	2657.74	{1728., 3617.}	35.6947	0.208116	newly 60 weeks cumul	7710.	6575.57	{4783., 7731.}	15.0554	0.0763766
41	newly 3 weeks spec	1055.	465.17	{329., 601.}	55.9081	0.366134	newly 3 weeks cumul	2675.	1238.88	{889., 1584.}	53.6867	0.344859	newly 5 weeks spec	964.	502.46	{367., 618.}	47.8776	0.291217	newly 5 weeks cumul	4723.	2231.24	{1617., 2831.}	52.758	0.335224	newly 59 weeks cumul	8244.	6399.65	{5183., 7053.}	22.372	0.113103
42	newly 3 weeks spec	812.	390.74	{315., 460.}	51.8793	0.322574	newly 3 weeks cumul	2241.	1139.68	{925., 1317.}	49.1441	0.298243	newly 5 weeks spec	727.	376.47	{306., 443.}	48.216	0.2901	newly 5 weeks cumul	3835.	1899.58	{1578., 2197.}	50.4673	0.309209	newly 54 weeks cumul	7356.	4997.23	{4223., 5496.}	32.0659	0.169774
43	newly 3 weeks spec	1345.	319.39	{120., 558.}	76.2535	0.698373	newly 3 weeks cumul	2361.	721.2	{288., 1201.}	69.4536	0.576095	newly 5 weeks spec	1603.	467.77	{157., 838.}	70.8191	0.61612	newly 5 weeks cumul	5687.	1591.01	{627., 2819.}	72.0237	0.623164	newly 64 weeks cumul	8914.	7067.42	{4808., 8986.}	21.184	0.112407
44	newly 3 weeks spec	783.	341.74	{272., 412.}	56.355	0.36666	newly 3 weeks cumul	2116.	972.34	{754., 1147.}	54.0482	0.34373	newly 5 weeks spec	810.	352.24	{277., 429.}	56.5136	0.367811	newly 5 weeks cumul	3802.	1677.73	{1297., 2041.}	55.8724	0.361177	newly 55 weeks cumul	7747.	5357.44	{4874., 5873.}	30.845	0.161329
45	newly 3 weeks spec	892.	467.66	{333., 623.}	47.5717	0.29579	newly 3 weeks cumul	2335.	1255.23	{923., 1685.}	46.2428	0.284398	newly 5 weeks spec	873.	494.71	{375., 658.}	43.3322	0.25844	newly 5 weeks cumul	4172.	2238.06	{1661., 2968.}	46.3552	0.284011	newly 59 weeks cumul	7795.	6225.72	{5084., 7240.}	20.1319	0.101733
46	newly 3 weeks spec	811.	433.34	{304., 551.}	46.5672	0.283387	newly 3 weeks cumul	2110.	1193.03	{872., 1520.}	43.4583	0.257997	newly 5 weeks spec	861.	449.91	{338., 550.}	47.7456	0.290071	newly 5 weeks cumul	3857.	2092.69	{1572., 2667.}	45.7431	0.27471	newly 57 weeks cumul	7935.	5983.96	{4996., 6619.}	24.5878	0.125021
47	newly 3 weeks spec	1061.	312.27	{197., 432.}	70.5683	0.552578	newly 3 weeks cumul	2374.	827.42	{524., 1125.}	65.1466	0.477271	newly 5 weeks spec	1104.	363.84	{231., 503.}	67.0435	0.500771	newly 5 weeks cumul	4682.	1537.35	{973., 2120.}	67.1647	0.50264	newly 59 weeks cumul	8780.	5931.51	{4627., 6986.}	32.4429	0.175395
48	newly 3 weeks spec	1085.	387.6	{185., 647.}	64.2765	0.493603	newly 3 weeks cumul	2381.	952.58	{469., 1586.}	59.9924	0.440577	newly 5 weeks spec	1180.	492.06	{214., 823.}	58.3	0.423618	newly 5 weeks cumul	4857.	1888.52	{908., 3144.}	61.1176	0.453564	newly 62 weeks cumul	8650.	6879.94	{5537., 8376.}	20.4726	0.105114
49	newly 3 weeks spec	1202.	324.83	{131., 602.}	72.9759	0.636864	newly 3 weeks cumul	2426.	788.09	{327., 1410.}	67.5148	0.546776	newly 5 weeks spec	1386.	438.06	{167., 831.}	68.3939	0.568346	newly 5 weeks cumul	5269.	1619.23	{675., 2920.}	69.2687	0.575268	newly 63 weeks cumul	8545.	6655.41	{4692., 8589.}	22.7065	0.122083
50	newly 3 weeks spec	1062.	309.88	{178., 480.}	70.8211	0.5639	newly 3 weeks cumul	2104.	777.6	{461., 1125.}	63.0418	0.4576	newly 5 weeks spec	1231.	382.69	{220., 573.}	68.9123	0.53555	newly 5 weeks cumul	4630.	1512.1	{908., 2282.}	67.3413	0.512444	newly 61 weeks cumul	8721.	6441.89	{5083., 7525.}	26.1336	0.136685
